# Chart, Don’t Theme. Why Reflexive Thematic Analysis is a Poor Fit for Scoping Reviews

**DOI:** 10.5334/pme.2670

**Published:** 2026-07-09

**Authors:** Anél Wiese, Lauren A. Maggio, Deirdre Bennett

**Affiliations:** 1 Medical Education Unit, School of Medicine, University College Cork, Ireland; 2 Department of Medical Education, University of Illinois Chicago, United States

## Abstract

Scoping reviews map the breadth and nature of a field. In medical education, however, an increasing number of scoping reviews report using thematic analysis on author-summarised results from included studies. This is a methodological mismatch. Reflexive thematic analysis (in the Braun & Clarke sense) is designed for rich primary qualitative datasets and has an explicitly interpretive logic. Scoping reviews, by contrast, work with secondary data and follow a descriptive, aggregative logic aimed at transparency and reproducibility. Mixing the two invites secondary re-interpretation and can mislead readers about what a scoping review can legitimately claim, while weakening the audit trail. We set out seven points where the two methods clash, covering purpose, epistemology, the level and unit of the data, how that data is collected, the analytic procedures, the outputs and the risk of bias. For each, we point to a fit-for-purpose alternative, whether structured charting, framework-based mapping, or qualitative content analysis. Our message practical. For credible evidence mapping, chart, don’t theme.

## Introduction

Scoping reviews have become a familiar way for medical education scholars to make sense of a body of literature, clarifying concepts, cataloguing interventions, identifying gaps, and signalling directions for future research [1,2]. The emphasis is on breadth and to show the lay of the land, not to generate new interpretations [3]. After a transparent search and selection process, the engine room of a scoping review is data charting. Reviewers pre-specify what they will extract (for example, aims, designs, settings, participants, and key reported findings), and then systematically chart those items into a standardised data charting template [4]. That template is not an end in itself; it underpins a descriptive synthesis that shows patterns and distributions (what exists, with whom, and in what forms) without re-interpreting the original studies.

Running alongside this tradition is reflexive thematic analysis in the Braun & Clarke tradition [5]. Reflexive thematic analysis is not just sorting information under topic headings; it is an interpretive method for making meaning from raw qualitative data [6]. Analysts generate themes through iterative engagement, writing and refining, asking what meaning is being made, and making their own positioning and decisions visible [7]. Credibility rests on principled subjectivity. More than one defensible theming can exist, provided the argument is coherent and made transparent through reflexivity about how it was produced [8]. Braun and Clarke distinguish reflexive thematic analysis from codebook approaches (such as template analysis) and coding-reliability thematic analysis, each of which carries different assumptions about researcher subjectivity and code development [9,10]. Our argument in this paper concerns reflexive thematic analysis specifically. Codebook approaches share features with the structured categorisation methods we recommend below and raise fewer of the concerns we outline (particularly the epistemological mismatch and the reflexivity requirement), though the level-of-data problem (applying any form of thematic analysis to compressed secondary summaries) still applies, and codebook approaches should be described precisely rather than conflated with reflexive thematic analysis.

So why do we see “we used thematic analysis” so often in scoping reviews [11]? It is a mix of history and habit, with some good intentions along the way. Early scoping guidance [12,13] sometimes used “thematic” as a loose synonym for grouping, and that wording stuck. Levac et al. in particular recommended that authors undertake “a thematic analysis” during stage 5 of their framework, describing it as resembling qualitative data-analytic techniques [13]. Levac et al. did not cite Braun and Clarke (2006) or any specific thematic analysis method. Read in context, they meant descriptive grouping and synthesis of charted data, not the reflexive, interpretive method that Braun and Clarke later codified. Yet the phrasing created an ambiguity that persists. Authors who cite Levac et al. as licence for “thematic analysis” in a scoping review may not realise that the intended procedure was closer to content analysis than to reflexive thematic analysis. Meanwhile, the charting stage (though central) was often under-described in methods sections, leaving authors to reach for a familiar label to explain how they organised results [13]. Add two more pressures. Researchers and reviewers sometimes look for “more depth”, and qualitative methods training often introduces thematic analysis as a versatile, reputable technique [14]. In that context, writing “we used thematic analysis” can feel like the safest way to signal that one has gone beyond mere counting without drifting into full meta-synthesis.

Software and scholarly cross-traffic compound the drift, by which we mean the way vocabulary and analytic habits travel across from neighbouring review traditions and settle where they do not belong. When results paragraphs from included papers are imported into qualitative data software [15], the interface invites coding. Codes invite categories; categories invite “themes”. Neighbouring review genres (e.g., qualitative evidence synthesis [16], thematic synthesis [17]) do use interpretive methods and it is easy to confuse their aims with those of a scoping review.

Our claim is about fit, not worth. Reflexive thematic analysis is a strong method, and so is scoping review. We argue only that reflexive thematic analysis does not suit the data and aims of a scoping review. Each earns its credibility in a different way, using different data and serving different purposes. One might object that reflexive thematic analysis is inherently flexible and can be adapted to any dataset. Braun and Clarke themselves, however, stress that reflexive thematic analysis requires data rich enough to sustain interpretive engagement and that themes are not just topic summaries but meaning-based patterns grounded in researcher flexibility [9,18]. They have explicitly criticised the production of superficial topic summary themes that lack interpretive depth, a problem they have documented across multiple fields [10,19]. When scoping reviews lift the language and procedures of reflexive thematic analysis and apply them to such materials, they blur that line between transparent mapping and interpretive argument. Readers can no longer tell whether they are looking at a map of what is there or an interpretation built from materials that were never designed for that purpose.

Our aim in this paper is practical, not punitive. We name this drift because it is common and understandable and because it quietly changes what a scoping review can claim. In the sections that follow, we set out seven predictable problems and, more importantly, workable alternatives that keep analysis fit-for-purpose.

**Key messages**

- **Different data, different goals:** Reflexive **thematic analysis** is designed for *primary* qualitative data; scoping reviews work with *secondary*, author-summarised data and map them descriptively.
- **Seven mismatches when reflexive thematic analysis is used in scoping reviews:** purpose, epistemology, the level and the unit of analysis, how data is collected, the procedures, the outputs, and the risk of bias.
- **Use fit-for-purpose approaches:** structured charting and framework-based mapping both work, as does qualitative content analysis.
- **Bottom line:** For credible, reproducible mapping, chart, don’t theme.

## Seven points of clash

At first glance, reflexive thematic analysis and scoping review analysis seem compatible because both involve looking for patterns in the data. But the similarities are superficial. Each method is designed for a different purpose and follows a distinct analytic logic. Reflexive thematic analysis is interpretive, whereas scoping review analysis is descriptive and aggregative. Before detailing the seven mismatches, we note one important caveat. All analysis involves some degree of interpretation, even deciding what counts as, for example, an intervention when charting requires judgement. The relevant distinction is not whether interpretation occurs but how much and on what warrant. Scoping review charting constrains interpretation through predefined categories and operational definitions. Reflexive thematic analysis embraces it as the primary analytic resource. Combining the two creates a methodological mismatch that can distort findings and mislead readers. Below, we delineate seven key points of incongruence.

### 1. Purpose

Reflexive thematic analysis generates interpretive insight and sometimes theory [5]. Scoping review maps the extent, nature, and distribution of evidence and signal gaps [12]. Interpretive aims exceed a scoping review’s descriptive mandate. Using reflexive thematic analysis pushes the review toward qualitative evidence synthesis, a different design with different methodological expectations [16]. The purpose gap is not a matter of effort or quality. Each method earns its credibility on its own data and for its own ends. A scoping review promises that anyone who follows the same search, selection, and charting rules could rebuild the descriptive map and land in roughly the same place. Reflexive thematic analysis makes a different promise, that a reader who followed the analyst’s reasoning on rich primary data would see why these themes make interpretive sense, even where another skilled analyst might read the same data and arrive somewhere else. Bolt a method built for the second promise onto a design built for the first, and the review starts answering a question it never set out to ask. The reader is then told what the literature might mean before being shown what it actually contains.

### 2. Epistemology

Reflexive thematic analysis is explicitly interpretivist/constructivist, which means that the researcher’s subjectivity is an analytic resource [8]. Scoping review is aggregative and procedural and credibility is built through auditable rules [4]. Importing reflexive subjectivity contradicts the review’s claim to reproducibility. The two stand on incompatible warrants. A scoping review rests its credibility on procedure, so a second team working the same rules should reach a substantively similar map. Reflexive thematic analysis rests its credibility on the analyst’s situated judgement, made defensible through reflexivity rather than through repeatability, which is why two careful analysts can read the same material and still differ. A review that borrows the interpretive moves of reflexive thematic analysis while still calling itself a scoping design quietly swaps one warrant for the other and tells the reader neither. What looks like a review can no longer be audited like one, because the grounds on which it asks to be believed have changed without notice.

### 3. Level and unit of data

Reflexive thematic analysis expects immersion in primary qualitative material (full transcripts, fieldnotes, documents) [7]. Scoping reviews have secondary author-summarised findings and structured metadata [20]. These are often stripped of the original participant voice and presented in compressed form, and vary widely in detail. Applying reflexive thematic analysis to summaries creates double interpretation (authors interpreted participants; reviewers then interpret the authors) without access to original meaning-making contexts. The unit of analysis shifts in the same move. Reflexive thematic analysis works on a qualitative dataset and treats the corpus of talk as one fabric to be read across [5]. A scoping review works on the study or publication, fixed by eligibility criteria, and each included paper is a bounded record rather than a strand of shared meaning [12]. When reviewers build themes across studies that differ in design and in how much they report, they read unlike units as if they were comparable, and the resulting pattern reflects what authors happened to write up rather than any meaning the participants made. Author-summarised findings rarely carry the richness that reflexive thematic analysis needs, because they are compressed and uneven in detail and stripped of the participant voice that gives an interpretation its warrant. Theming such material does not deepen the map. It loosens the link between what a study reported and what the review claims about it.

### 4. Data collection

The two methods stand on different kinds of data, and the difference starts before any coding begins. Reflexive thematic analysis works with data the researcher has generated for the question at hand. The interviews or focus groups are designed around the aims of the study, run by people who can follow up, probe, and return to the field when an early reading raises a new line of enquiry [5]. A scoping review has no such control over its raw material. It maps a body of literature that already exists, so the data are whatever previous authors chose to report, written up for their own purposes and to their own depth [12]. Once selection and extraction are finalised, the reviewer cannot go back to the field to fill a gap a developing theme has opened up. The dataset is closed, and it was never built for this. Reflexive thematic analysis assumes a corpus generated to be read deeply, while the scoping review hands the analyst a set of second-hand summaries and asks the same depth of them. The data simply do not stretch that far.

### 5. Procedures

Reflexive thematic analysis involves iterative coding, theme development, and active reflexivity, often cycling back to raw data to deepen interpretations [21]. Scoping reviews follow a structured charting process of predefined items, piloting for consistency, and descriptive synthesis with limited scope for emergent categories [22]. Inventing thematic analysis-style codes and themes from short study summaries generates fragile structures that cannot be audited against an original dataset. The two workflows pull in opposite directions. Reflexive thematic analysis wants the analyst to keep moving between the codes and the raw material, loosening and tightening the reading as understanding builds. A scoping review wants the opposite, a charting form settled in advance and applied the same way to every study, so that the process can be checked. Run the reflexive workflow over study summaries, and the safeguards that make charting auditable fall away, because there is no original dataset to return to and no fixed frame to test the coding against. The themes that result look worked through, yet nothing underneath them can be re-run by another reviewer. That is the part that should worry an editor, since it is precisely the auditability a scoping review is meant to offer.

### 6. Outputs

Reflexive thematic analysis generates themes supported by illustrative quotes from primary data [23]. Scoping review produces categories/domains, frequencies, counts, cross-tabulations, evidence maps and structured descriptive narrative summaries [3]. Thematic analysis-like “themes” in a scoping review read like qualitative findings without data to support them. An output carries the marks of the method that made it. A reflexive theme is meant to be a meaning-based pattern, earned by reading across rich material and shown to the reader through extracts that let them judge the reading for themselves. A scoping review output is a different object, a count, a cross-tabulation, a coverage map, a structured account of what the field holds and where it thins out. When a scoping review presents named themes drawn from author summaries, it offers the form of an interpretive finding with none of the primary data that would let a reader test it. Braun and Clarke have made this complaint themselves, that themes are too often topic summaries dressed as analysis [8]. The version that worries us is worse, since the topic summary is built from summaries already made by other authors, one more remove from anything a participant said.

### 7. Bias risk

In reflexive thematic analysis, theme development depends on interpretive engagement with the richness of the material [20,24]. In scoping review, charting mitigates bias via consistent variables and explicit rules [25]. Studies with richer reported findings may contribute disproportionately to themes not because they are substantively more important but because they provide more material for interpretive engagement, which is a data-richness confound. The confound runs the wrong way for a map that is supposed to weigh the field evenly. A study that happens to be written up in detail offers more for the analyst to work with, so it pulls harder on the developing themes, while a terse study reporting the same finding barely registers. The pattern then tracks how much authors chose to report rather than what the literature actually shows, and the studies that report least, often the smaller or less resourced ones, drop out of view. Charting guards against this by recording the same fields for every study, even where the answer is “not reported”, so absence stays visible. Theming has no such guard. It rewards the talkative paper and quietly discounts the rest.

When reflexive thematic analysis is applied to scoping review data, the product is neither a genuine thematic analysis (insufficient data richness) nor a methodologically sound scoping review (exceeds descriptive remit). The outcome is a hybrid that is less transparent, less reproducible, and more prone to over-interpretation than either approach done properly.

**Quick diagnostic: Are you drifting into reflexive thematic analysis?**

- You are crafting interpretive theme titles (e.g., becoming a professional through feedback) from study summaries, not primary data.
- Your inclusion criteria or search changed mid-way because a developing theme looked promising.
- Two analysts produced different themes, and you resolved them by narrative compromise rather than rule refinement.
- Your results read like qualitative findings, with little in the way of counts, maps or tables.
- You cite Braun & Clarke yet present no primary qualitative dataset or data extracts.

If any ring true, step back. Either: a) re-specify your scoping analysis as charting/mapping/content analysis, or b) split into a scoping review plus a separate qualitative evidence synthesis.

## Fit-for-purpose alternatives

When the aim is to analyse scoping review data in a methodologically sound way, several approaches align with the review’s purpose and epistemological stance. We consolidate and operationalise three established, fit-for-purpose approaches (structured charting, framework-based mapping, and qualitative content analysis) already described in scoping review guidance and methods literature [4,20]. Each preserves the descriptive, mapping intent of a scoping review while still allowing meaningful organisation of information.

### Structured charting and categorisation

Structured charting entails specifying a predefined set of data items closely linked to the review questions and systematically extracting them from every included study [4]. Each field is defined clearly in the protocol (e.g., learner group, educational strategy, outcome type, study design), with coding guidance for different formats. The same fields are populated for each study, even if some remain “not reported” to ensure compatibility and aggregation. Categories may be pre-specified (deductive) or refined slightly after a pilot phase to improve clarity and consistency.

**Use when:** Your goal is to map the scope and characteristics of the literature in a way that permits easy comparison and summary. It is ideal for answering “what exists, how much, where, and with what reported features/outcomes?” questions. This approach is particularly useful for large, heterogeneous datasets and when the intended outputs include frequency counts, summary tables, or coverage maps.

**Steps:**

1. Derive data fields from your questions (context, participants, intervention/exposure, comparators, outcomes, design, etc.).
2. Draft operational definitions; pilot on 8–10 studies.
3. Refine fields for clarity and mutual exclusivity.
4. Calibrate reviewers (two authors double-chart a subset of studies to assess consistency and resolve discrepancies by rule refinement).
5. Summarise descriptively.
6. Present evidence maps/tables that map coverage and gaps; write a structured narrative keyed to the map.

**Strengths:** Transparent, reproducible, scalable.

**Watch-outs:** Resist the temptation to invent interpretive labels; keep categories observable.

#### Stock wording (Methods)

*We developed and piloted a charting form aligned to our review questions (e.g., what is the setting, learner group, modality, outcome type, study design?). After piloting on 10 studies and refining operational definitions, we applied the final form to all included studies. We summarised charted data descriptively (frequencies, cross-tabulations) and generated evidence maps*.

#### Example

Park et al. coded study characteristics including facilitation mode (e.g., videoconferencing), learner outcomes measured (e.g., knowledge improvement), and study design and then reported frequencies and distribution across the sample [26].

### Framework-based analysis

Framework-based analysis uses an existing model, taxonomy, or policy framework as the organising structure for the extracted data [27]. Findings from each study are mapped to one or more framework domains or categories using clear operational definitions. Mapping can be done fully deductively (fitting all data into pre-existing slots) or adaptively (adding new categories when data do not fit). Note that framework analysis [27] was originally developed for primary qualitative data, but has been adapted for secondary/review data [28] making it an example of a method that can cross the primary/secondary boundary when properly specified, unlike reflexive thematic analysis.

**Use when:** A well-established conceptual model is directly relevant to your review question. Especially when you aim to benchmark the literature against an existing standard (e.g., curriculum design framework, competency framework, Kirkpatrick evaluation levels) or identify gaps in the literature relative to a model (e.g., which domains are underrepresented).

**Steps:**

1. Choose a framework justified by the aim (cite it and define all domains clearly).
2. Translate domains into coding rules with examples and notes for boundary cases.
3. Pilot the mapping on a small subset (e.g., <10 of total publications); refine rules and document decisions.
4. Map each study to one or more domains; record uncertainties and “not applicable”.
5. Summarise distributions and gaps by domain and discuss.

**Strengths:** Actionable for educators and policymakers; easy to compare across contexts.

**Watch-outs:** Avoid forcing data into domains that do not fit. Use an “other/unclear” bucket transparently.

#### Stock wording (Methods)

*We mapped extracted findings to the [Framework] domains, using operational definitions developed a priori and refined during piloting. Two reviewers independently applied the final domains to all studies; disagreements were resolved through discussion and prompted rule refinement. We summarised coverage and gaps by domain*.

#### Example

Meyer et al. used the Ottawa Conference Criteria for Good Assessment as a framework to categorise and map included studies [29].

### Qualitative content analysis

Qualitative content analysis involves systematically coding extracted text for the explicit presence or absence of concepts, terms, or features and counting their frequency across the dataset [20]. Coding can be purely deductive (based on a predefined list of concepts), inductive (allowing new categories to be extracted from the data), or a hybrid of both approaches, but the goal remains descriptive rather than interpretive [30,31]. Although both Elo and Kyngäs and Hsieh and Shannon note that latent content can be analysed, qualitative content analysis as we apply it here does not routinely interpret latent meaning, staying focused on what is explicitly reported [30,31].

**Use when:** You want to quantify reporting patterns across the included studies. The approach is especially useful for large corpora, for audiences that value numerical summaries, and when frequency patterns are meaningful.

**Steps:**

1. Draft a coding frame (deductive from questions/framework; inductive categories followed only if explicit in the text).
2. Pilot and refine the frame for clarity, mutual exclusivity, and exhaustiveness.
3. Code all extracted items; track multi-labels where appropriate and track co-occurrence.
4. Summarise counts and co-occurrence descriptively.
5. Visualise with bar charts, heat maps, or evidence gap maps.

**Strengths:** Adds nuance without drifting into interpretive claims; auditable.

**Watch-outs:** Keep strictly manifest; if you must infer, you have left content analysis.

#### Stock wording (Methods)

*We conducted a descriptive qualitative content analysis of extracted findings, coding explicit mentions of predefined concepts. Following piloting and refining the coding frame, we applied it across the corpus and summarised frequencies and co-occurrences*.

#### Example

Bochatay et al. coded extracted study details for the presence/absence of predefined features (e.g., simulation modality, professional mix, teamwork skills targeted), tallied frequencies, and presented descriptive statistics to map trends [32].

### Terminology and boundary conditions

In everyday writing, it can seem reasonable to say studies were “grouped into themes”, but in methods reporting, that phrasing signals an interpretive approach that is inappropriate for scoping reviews. In scoping reviews, use terminology like *category, domain*, or *topic grouping*. If using the word “theme” colloquially, say so in the methods, for example: *We use “theme” here to mean descriptive grouping, not reflexive thematic analysis*.

Several boundary conditions deserve note:

- **Two-stage projects:** It is appropriate to pair a scoping review (to map the field) with a separate qualitative evidence synthesis (to interpret a subset). Keep designs, methods, and outputs clearly separated and labelled.
- **Narrative synthesis ≠ reflexive thematic analysis:** Narrative or structured descriptive synthesis can integrate and explain patterns without adopting thematic analysis’s interpretive commitments.
- **Colloquial “themes”:** If your journal insists on “themes” as a word for groupings, define your terms explicitly as descriptive categories to avoid implying reflexive thematic analysis.

**Salvage plan if you already used “thematic analysis”**

1. **Relabel accurately**. If your procedure was grouping/charting, rename outputs “categories” or “domains”, not “themes.
2. **Add missing charting**. Define variables post hoc; re-extract; provide tables/maps.
3. **Move interpretive bits**. Reframe them as implications or hypotheses for future study, not as findings.
4. **Consider a Stage-2 qualitative evidence synthesis**. If you genuinely have an interpretive contribution, propose (or conduct) a separate qualitative synthesis with appropriate methods (e.g., thematic synthesis, meta-ethnography).
5. **Be transparent**. Acknowledge the correction in the Methods and justify the re-specification.

## Guidance for authors, reviewers, and editors

Methodological fit in scoping reviews is not a matter of taste. It follows from how reviews justify their claims. Scoping reviews can earn credibility by making their procedures visible and repeatable. A documented search and explicit selection rules, together with operational definitions, let another team work the same corpus and arrive at substantively similar tables and descriptive summaries [33]. Reflexive thematic analysis, by contrast, earns credibility through principled subjectivity, the analysts’ situated judgement, made transparent through reflexivity and argumentation, and therefore permits more than one legitimate reading of a primary qualitative dataset [34]. Both approaches are defensible, but they warrant claims differently. Confusion sets in when a review borrows reflexive thematic analysis’s interpretive moves while still presenting itself as a scoping design. The result is a hybrid that looks like a review but cannot be audited like one.

For authors, the practical implication is straightforward. Begin with the aim. If the question is “what exists, where, and with what features?”, the analysis should remain descriptive and aggregative. Chart what studies report, count and cross-tabulate those features, and narrate coverage and gaps [4]. This is not a lesser standard; it is a different one. It asks you to invest effort in rules (a piloted charting form with clear, mutually exclusive categories) rather than in interpretive theme development. Where interpretive insight is genuinely needed (where the contribution depends on reading across meaning rather than cataloguing features), the design should be named and conducted as a qualitative evidence synthesis and judged on those terms.

For reviewers and editors, the task is to guard the warrant. When a manuscript labelled as a scoping review presents interpretive “themes” derived from author-summarised results, it has shifted from procedural reproducibility to principled subjectivity without acknowledging the move. That shift is not a fatal flaw if the authors are willing to re-specify the analysis. In practice, this means relabelling outputs as categories or domains, providing the charting fields and their operational definitions, and presenting counts, cross-tabs, or an evidence map to render decisions auditable. If the interpretive layer is central to the contribution, it is more honest (and more useful to readers) to reframe the piece as a qualitative synthesis and align it with the appropriate standards.

Editors and researchers sometimes worry that insisting on charting and mapping will “flatten” the field. The opposite is true. Clean descriptive work clarifies what is present and, just as much, what is missing. It creates a stable platform for theory-building work to stand on, and it prevents the field from mistaking eloquent summaries for new analysis. Scoping reviews should not attempt to do everything. They should do their one thing well, making the terrain visible and reproducible.

**Rapid appraisal checklist**

- **Aim:** Does the manuscript’s aim/objective/question read as a mapping/description rather than an interpretive explanation?
- **Preparation:** Are charting data items or framework domains defined a priori and applied consistently? Are operational definitions visible (with note of piloting/calibration)?
- **Data-method fit:** Are secondary summaries analysed via charting/framework mapping/content analysis (not reflexive thematic analysis)?
- **Outputs:** Are results presented as descriptive categories, counts, or maps rather than interpretive “themes”?
- **Terminology:** If “thematic analysis” is claimed, is it explicitly not reflexive thematic analysis? If it is a reflexive thematic analysis, advise reframing as a qualitative evidence synthesis or relabelling as descriptive grouping/content analysis.
- **Reporting:** Are PRISMA-ScR items met, and are analytic choices transparent and reproducible?

## Scope and caveats

We focus specifically on reflexive thematic analysis (Braun & Clarke) [5,8,9]. We do not evaluate other “thematic” approaches designed for secondary data (e.g., thematic synthesis, meta-aggregation) that are legitimate within qualitative evidence syntheses [16]. Our arguments should not be over-generalised to those methods or to qualitative evidence synthesis designs.

We write from (and for) medical education, but the core issue (misaligning interpretive methods with scoping review aims and data) is cross-disciplinary [35]. Disciplines that routinely use scoping reviews may face the same drift and can adopt our recommendations. This is a methodological argument, not a prevalence study. We do not quantify how often reflexive thematic analysis is used in scoping reviews or measure its effects on review quality; examples are illustrative rather than exhaustive. Future work could usefully examine the prevalence of thematic analysis claims in published scoping reviews and assess whether analytic procedures align with the labels used. Data charting inevitably involves reviewer judgement. Piloting the data charting template, refining rules, and keeping an audit trail mitigate (but do not eliminate) subjectivity. Reproducibility still depends on clear reporting by review teams. In small or highly heterogeneous literatures, descriptive mapping may look sparse. Our recommendation is to resist the urge to inflate claims. Name the gap and move on. However, this approach may limit immediate practice implications. We do not provide a decision framework for when to shift to a qualitative evidence synthesis; this is a question future methodological work could usefully address. Standards and language differ across JBI, PRISMA-ScR, and other guidance [33,36]. Readers will need to adapt our recommendations to local editorial expectations and disciplinary conventions.

## Conclusion

Reflexive thematic analysis has an important place in qualitative research, but that place is not at the analytic core of a scoping review. Scoping reviews should chart and map the field against the review’s descriptive purpose. Applying reflexive thematic analysis to extracted findings from published studies imports an interpretive logic that conflicts with the epistemology and procedural standards of scoping review methodology. The result is a methodologically incongruent hybrid, neither a true thematic analysis nor a sound scoping review.

By committing to fit-for-purpose analysis methods, authors can avoid the seven pitfalls outlined here. This is not a call for mechanical scoping reviews devoid of insight, but an encouragement to respect scoping reviews’ distinct contribution within the evidence-synthesis ecosystem.

So our message is to chart, not theme. Charting done well is the methodologically correct choice rather than a lesser one, and it produces outputs that readers across education and policy can trust and use.

## References

[1] Maggio LA, Costello JA, Norton C, Driessen EW, Artino AR. Knowledge syntheses in medical education: A bibliometric analysis. Perspect Med Educ. 2021;10. DOI: 10.1007/S40037-020-00626-9 PMC7580500 33090330

[2] Mak S, Thomas A. An Introduction to Scoping Reviews. J Grad Med Educ. 2022;14:561–564. DOI: 10.4300/JGME-D-22-00620.1 36274758 PMC9580317

[3] Munn Z, Peters MDJ, Stern C, Tufanaru C, McArthur A, Aromataris E. Systematic review or scoping review? Guidance for authors when choosing between a systematic or scoping review approach. BMC Med Res Methodol. 2018;18:143. DOI: 10.1186/s12874-018-0611-x 30453902 PMC6245623

[4] Peters MDJ, Marnie C, Tricco AC, Pollock D, Munn Z, Alexander L, et al. Updated methodological guidance for the conduct of scoping reviews. JBI Evid Synth. 2020;18:2119. DOI: 10.11124/JBIES-20-00167 33038124

[5] Braun V, Clarke V. Thematic Analysis: A Practical Guide. SAGE Publications Ltd; 2021. DOI: 10.4135/9781036232078.n9

[6] Coates WC, Jordan J, Clarke SO. A practical guide for conducting qualitative research in medical education: Part 2—Coding and thematic analysis. AEM Educ Train. 2021;5:e10645. DOI: 10.1002/aet2.10645 34585038 PMC8457700

[7] Kiger ME, Varpio L. Thematic analysis of qualitative data: AMEE Guide No. 131. Med Teach. Taylor & Francis. 2020;42:846–854. DOI: 10.1080/0142159X.2020.1755030 32356468

[8] Braun V, Clarke V. One size fits all? What counts as quality practice in (reflexive) thematic analysis? Qual Res Psychol. Routledge. 2021;18:328–352. DOI: 10.1080/14780887.2020.1769238

[9] Braun V, Clarke V. Toward good practice in thematic analysis: Avoiding common problems and be(com)ing a knowing researcher. Int J Transgender Health. Taylor & Francis. 2023;24:1–6. DOI: 10.1080/26895269.2022.2129597 PMC9879167 36713144

[10] Braun V, Clarke V, Hayfield N, Terry G. Thematic Analysis. Handb Res Methods Health Soc Sci [Internet]. Singapore: Springer; 2019 [cited 2026 Mar 1]. p. 843–860. DOI: 10.1007/978-981-10-5251-4_103

[11] Maggio LA, Larsen K, Thomas A, Costello JA, Artino AR. Scoping reviews in medical education: A scoping review. Med Educ. 2021;55:689–700. DOI: 10.1111/medu.14431 33300124 PMC8247025

[12] Arksey H, O’Malley L. Scoping studies: towards a methodological framework. Int J Soc Res Methodol. Routledge. 2005;8:19–32. DOI: 10.1080/1364557032000119616

[13] Levac D, Colquhoun H, O’Brien KK. Scoping studies: advancing the methodology. Implement Sci. 2010;5:69. DOI: 10.1186/1748-5908-5-69 20854677 PMC2954944

[14] Nowell LS, Norris JM, White DE, Moules NJ. Thematic Analysis: Striving to Meet the Trustworthiness Criteria. Int J Qual Methods. 2017;16:1609406917733847. SAGE Publications Inc. DOI: 10.1177/1609406917733847

[15] Dhakal K. NVivo. J Med Libr Assoc JMLA. 2022;110:270–272. DOI: 10.5195/jmla.2022.1271 35440911 PMC9014916

[16] Flemming K, Noyes J. Qualitative Evidence Synthesis: Where Are We at? Int J Qual Methods. SAGE Publications Inc. 2021;20:1609406921993276. DOI: 10.1177/1609406921993276

[17] Thomas J, Harden A. Methods for the thematic synthesis of qualitative research in systematic reviews. BMC Med Res Methodol. 2008;8:45. DOI: 10.1186/1471-2288-8-45 18616818 PMC2478656

[18] Braun V, Clarke V. Reflecting on reflexive thematic analysis. Qual Res Sport Exerc Health. 2019;11:589–597. Routledge. DOI: 10.1080/2159676X.2019.1628806

[19] Morriss L. Themes do not emerge. An editor’s reflections on the use of Braun and Clarke’s thematic analysis. Qual Soc Work. 2024;23:745–749. SAGE Publications. DOI: 10.1177/14733250241277355

[20] Pollock D, Peters MDJ, Khalil H, McInerney P, Alexander L, Tricco AC, et al. Recommendations for the extraction, analysis, and presentation of results in scoping reviews. JBI Evid Synth. 2023;21:520. DOI: 10.11124/JBIES-22-00123 36081365

[21] Braun V, Clarke V. Using thematic analysis in psychology. Qual Res Psychol. 2006;3:77–101. Routledge. DOI: 10.1191/1478088706qp063oa

[22] Colquhoun HL, Levac D, O’Brien KK, Straus S, Tricco AC, Perrier L, et al. Scoping reviews: time for clarity in definition, methods, and reporting. J Clin Epidemiol. 2014;67:1291–1294. DOI: 10.1016/j.jclinepi.2014.03.013 25034198

[23] Braun V, Clarke V. Conceptual and design thinking for thematic analysis. Qual Psychol. US: Educational Publishing Foundation; 2022;9:3–26. DOI: 10.1037/qup0000196

[24] Dixon-Woods M, Agarwal S, Jones D, Young B, Sutton A. Synthesising qualitative and quantitative evidence: a review of possible methods. J Health Serv Res Policy. 2005;10:45–53. DOI: 10.1177/135581960501000110 15667704

[25] Daudt HM, van Mossel C, Scott SJ. Enhancing the scoping study methodology: a large, inter-professional team’s experience with Arksey and O’Malley’s framework. BMC Med Res Methodol. 2013;13:48. DOI: 10.1186/1471-2288-13-48 23522333 PMC3614526

[26] Park JO, Lee-Jayaram J, Sato E, Eto Y, Kahili-Heede M, Hirayama K, et al. A scoping review of remote facilitation during simulation-based healthcare education. BMC Med Educ. 2023;23:592. DOI: 10.1186/s12909-023-04551-3 37605196 PMC10464104

[27] Gale NK, Heath G, Cameron E, Rashid S, Redwood S. Using the framework method for the analysis of qualitative data in multi-disciplinary health research. BMC Med Res Methodol. 2013;13:117. DOI: 10.1186/1471-2288-13-117 24047204 PMC3848812

[28] Dixon-Woods M. Using framework-based synthesis for conducting reviews of qualitative studies. BMC Med. 2011;9:39. DOI: 10.1186/1741-7015-9-39 21492447 PMC3095548

[29] Meyer EG, Chen HC, Uijtdehaage S, Durning SJ, Maggio LA. Scoping Review of Entrustable Professional Activities in Undergraduate Medical Education. Acad Med. 2019;94:1040. DOI: 10.1097/ACM.0000000000002735 30946134

[30] Elo S, Kyngäs H. The qualitative content analysis process. J Adv Nurs. 2008;62:107–115. DOI: 10.1111/j.1365-2648.2007.04569.x 18352969

[31] Hsieh H-F, Shannon SE. Three Approaches to Qualitative Content Analysis. Qual Health Res. 2005;15:1277–1288. SAGE Publications Inc. DOI: 10.1177/1049732305276687 16204405

[32] Bochatay N, Ju M, O’Brien BC, van Schaik SM. A Scoping Review of Interprofessional Simulation-Based Team Training Programs. Simul Healthc. 2025;20:33. DOI: 10.1097/SIH.0000000000000792 38526045 PMC11776884

[33] Tricco AC, Lillie E, Zarin W, O’Brien KK, Colquhoun H, Levac D, et al. PRISMA Extension for Scoping Reviews (PRISMA-ScR): Checklist and Explanation. Ann Intern Med. American College of Physicians; 2018;169:467–473. DOI: 10.7326/M18-0850 30178033

[34] Byrne D. A worked example of Braun and Clarke’s approach to reflexive thematic analysis. Qual Quant. 2022;56:1391–1412. DOI: 10.1007/s11135-021-01182-y

[35] Pham MT, Rajić A, Greig JD, Sargeant JM, Papadopoulos A, McEwen SA. A scoping review of scoping reviews: advancing the approach and enhancing the consistency. Res Synth Methods. 2014;5:371–385. DOI: 10.1002/jrsm.1123 26052958 PMC4491356

[36] Peters M, Godfrey C, McInerney P, Baldini S, Khalil H, Parker D. Chapter 11: Scoping reviews. Joanna Briggs Inst Rev Man. The Joanna Briggs Institute; 2021.

